# Dental Fees

**Published:** 1881-04

**Authors:** 


Dental Fees.—" The laborer is worthy of his hire." The majority of dentists have educated the people not to expect fees for failures, examinations, opinions and broken engagements. The laity will weigh our ability as we value our professional services, provided we measure up to the *required standard.* It is a duty we owe to each other to have liberal remuneration for our services, and we will only have to educate our patients to appreciate our skill by charging round fees. All doctors have to be charitable to the poor. There is room for all, and let us not under bid—he who does so rates himself lower than his competitor. A mistake of the dental profession is to make good all mistakes. This is wrong, as all dentists and physicians should render their services honorably, and to the best of their ability, then if the tooth is lost or the patient dies they should be remunerated. We quote the following from the San Francisco Post :

"DR. YOUNGER, the Dentist, yesterday recovered $435 against H. M. Franklin and wife for professional work on the teeth of Mrs. Franklin. The complainant alleged that the defendant, Mrs. H. M. Franklin, by and with the consent of her husband, employed plaintiff to perform certain professional services as a dentist upon her teeth, and that in pursuance of such employment, between July 6, 1877, and September 5, 1877, plaintiff performed professional services, and furnished material for the same ; that the defendant failed to keep appointments made by her in the course of the services, whereby plaintiff lost valuable time, and was prevented from attending to other professional business ; that the services were worth $435. The particulars of the plaintiff's demands were as follows : one half hour examination, $8 ; two hours preparing and filling right upper lateral incisor, $30 ; one-quarter hour treating lower second bicuspid, $3 ; one-quarter hour same, $4 ; two and one-half hours filling right upper central incisor, and treating, $37.50; three and one-quarter hours, filling left upper lateral incisor, $49 ; three and one-quarter hours filling right upper canine, $45 ; three hours preparing cavities in lateral incisor and canine, $45 ; two and one-quarter hours treating and preparing cavity in right lower bicuspid, $32 ; two hours and forty minutes filling right lower bicuspid, $38 ; three hours filling right lower first bicuspid, $45; one and one-half hours finishing fillings and polishing teeth, $22.50 ; seven and one-half hours lost time, $75.

